# Metformin prevents the effects of *Pseudomonas aeruginosa* on airway epithelial tight junctions and restricts hyperglycaemia‐induced bacterial growth

**DOI:** 10.1111/jcmm.12784

**Published:** 2016-02-02

**Authors:** Wishwanath R. A. Patkee, Georgina Carr, Emma H. Baker, Deborah L. Baines, James P. Garnett

**Affiliations:** ^1^ Institute for Infection and Immunity St George's, University of London London UK; ^2^ Institute of Cellular Medicine Newcastle University Newcastle‐upon‐Tyne UK

**Keywords:** glucose, *P. aeruginosa*, airway epithelium, tight junctions, metformin, AMPK, claudin‐1, occludin, respiratory infection, diabetes

## Abstract

Lung disease and elevation of blood glucose are associated with increased glucose concentration in the airway surface liquid (ASL). Raised ASL glucose is associated with increased susceptibility to infection by respiratory pathogens including *Staphylococcus aureus and Pseudomonas aeruginosa*. We have previously shown that the anti‐diabetes drug, metformin, reduces glucose‐induced *S. aureus* growth across *in vitro* airway epithelial cultures. The aim of this study was to investigate whether metformin has the potential to reduce glucose‐induced *P. aeruginosa* infections across airway epithelial (Calu‐3) cultures by limiting glucose permeability. We also explored the effect of *P. aeruginosa* and metformin on airway epithelial barrier function by investigating changes in tight junction protein abundance. Apical *P. aeruginosa* growth increased with basolateral glucose concentration, reduced transepithelial electrical resistance (TEER) and increased paracellular glucose flux. Metformin pre‐treatment of the epithelium inhibited the glucose‐induced growth of *P. aeruginosa*, increased TEER and decreased glucose flux. Similar effects on bacterial growth and TEER were observed with the AMP activated protein kinase agonist, 5‐aminoimidazole‐4‐carboxamide ribonucleotide. Interestingly, metformin was able to prevent the *P. aeruginosa*‐induced reduction in the abundance of tight junction proteins, claudin‐1 and occludin. Our study highlights the potential of metformin to reduce hyperglycaemia‐induced *P. aeruginosa* growth through airway epithelial tight junction modulation, and that claudin‐1 and occludin could be important targets to regulate glucose permeability across airway epithelia and supress bacterial growth. Further investigation into the mechanisms regulating metformin and *P. aeruginosa* action on airway epithelial tight junctions could yield new therapeutic targets to prevent/suppress hyperglycaemia‐induced respiratory infections, avoiding the use of antibiotics.

## Introduction

The airway epithelium is covered by a thin layer of surface liquid (ASL), which is vital for maintaining a healthy respiratory tract. The ASL contains many potential growth substrates for bacterial growth including glucose. Glucose concentration in human ASL is normally ~0.4 mM, 12.5 times lower than that of blood 1. Airway surface liquid glucose concentrations are elevated in individuals with respiratory disease including acute viral rhinitis 2, chronic rhinosinitis 3, chronic obstructive pulmonary disease 4 and cystic fibrosis 1. Airway surface liquid glucose concentrations are also increased in experimental and diabetic hyperglycaemia 2, 5 and are further increased in individuals with both respiratory disease and diabetes mellitus 1, 6. Such elevations in ASL glucose are associated with increased susceptibility to infection, particularly with *S. aureus* and *Pseudomonas aeruginosa* 4, 6, 7.

We developed an *in vitro* model of airway glucose homoeostasis and showed that ASL glucose concentration is the net effect of the paracellular diffusion of glucose from the blood/interstitial fluid across the airway epithelium into ASL and its removal from ASL by epithelial uptake through facilitative glucose transporters 8, 9, 10, 11. We showed that paracellular glucose diffusion was increased not only by elevating basolateral glucose concentrations (mimicking hyperglycaemia of blood/interstitial fluid), but also by inflammation. In human epithelial cell monolayers, we found that application of either apical pro‐inflammatory cytokines 9 or apical bacterial pathogens 12, 13 increased paracellular glucose flux, increasing glucose concentrations in ASL. Under pro‐inflammatory conditions, airway epithelial glucose uptake through apical GLUTs is elevated, but this is insufficient to compensate for the increased paracellular glucose leak and does not prevent elevation of ASL glucose concentrations 9. Control of paracellular glucose movement thus appears to be the main rate‐limiting step for maintaining low ASL glucose concentrations.

We have previously shown that pre‐treatment of airway epithelial monolayers with the anti‐diabetes drug, metformin, attenuated the effects of *S. aureus* on paracellular glucose flux and reduced bacterial growth in ASL by restricting glucose availability 12. Reducing glucose permeability could therefore be a therapeutic target in the prevention and treatment of respiratory *S. aureus* infections.

Epithelial barrier dysfunction during bacterial infections is characterized by alterations in tight junction protein abundance. Tight junctions, the most apically located of the intercellular junctional complexes, regulate the passage of fluid, ions, macromolecules and inflammatory cells through the paracellular space between epithelial cells. Tight junctions are formed by three classes of integral membrane proteins: claudins, occludins and junctional adhesion molecules. Directly underneath tight junctions, are another specialized cell–cell contact domain, adherens junctions, which are mainly composed of cadherin family members. The composition of these protein complexes regulates epithelial permeability to solutes and ions. Factors that alter the abundance and localization of specific tight junction proteins within this complex could therefore alter glucose permeability.

The aim of this study was to determine whether metformin also has the potential to reduce glucose‐induced *P. aeruginosa* infections across airway epithelial cultures by limiting glucose permeability. We used the adenocarcinoma cell line, Calu‐3, derived from human tracheobronchial submucosal glands, which form a heterogeneous population of mucus (mucin‐secreting) and serous (fluid‐secreting) cell types 14, 15. Calu‐3 cells secrete a fluid rich in antimicrobial secretions to suppress bacterial growth 16 and form polarized airway epithelial monolayers with functional tight junctions expressing many of the integral membrane proteins found *in vivo* 17, 18. We explored the effect of *P. aeruginosa* and metformin on airway epithelial barrier function by investigating changes in tight junction protein abundance.

## Materials and methods

### Airway epithelial cell culture

The human adenocarcinoma‐derived cell line, Calu‐3 (from ATCC), was grown in Eagle's minimal essential medium plus 10% fetal calf serum, 2 mM L‐Glutamine, 100 units/ml penicillin, 100 μg/ml streptomycin, and 1% non‐essential amino acids (Sigma‐Aldrich, Poole, UK) and incubated in humidified air containing 5% CO_2_ at 37°C. Calu‐3 cells were seeded onto clear Costar Transwell inserts (0.45‐μm pore size) at 250,000 cells/cm^2^ to form confluent polarized monolayers, as previously described 19. Experiments were carried out 10–14 days post‐seeding. Twenty‐four hours before the experiment, monolayers were washed to remove the mucus layer and placed in Krebs salt solution consisting of 115 mM NaCl, 5 mM KCl, 25 mM NaHCO_3_, 1 mM MgCl_2_, 1 mM CaCl_2_ and 5 mM D‐glucose (equilibrated with 5% CO_2_ to pH 7.4) to remove antibiotics and components of the media that could influence bacterial growth.

Calu‐3 monolayers were pre‐treated with (or without) metformin (0.02 or 1 mM) or 5‐aminoimidazole‐4‐carboxamide ribonucleotide (AICAR; 0.5 mM), added to the basolateral medium 18 hrs prior to apical inoculation with *P. aeruginosa*. Metformin was applied to the basolateral membrane to mimic the situation *in vivo*, in which metformin would reach the airway epithelium *via* the blood/interstitial fluid. Calu‐3 cells and other bronchial cell types express organic cation transporter 3 on the basolateral membrane which is known to facilitate metformin uptake 20, 21, 22.

Using a chopstick style epithelial voltohmmeter (WPI, Hitchin, UK), Transepithelial electrical resistance (TEER) measurements were made, a widely accepted quantitative technique to measure the presence of tight junctions, the major component of epithelial cell barrier integrity in cell culture models of epithelial monolayers. Transepithelial electrical resistance measurements quantify the transport of ions across the cells with paracellular ion transport representing the most substantial contribution to such readings. An intact cellular barrier displays high TEER values. Loss of barrier integrity leads to decreased TEER values.

### Airway epithelia – *P. aeruginosa* co‐culture

A single colony of *P. aeruginosa* strain PA01 was incubated overnight at 37°C in RPMI media (Life Technologies, Paisley, UK) and diluted with glucose‐free RPMI to produce a culture of approximately 10^7^ colony forming units (CFU) per ml. 100 μl of PA01 culture (1 × 10^6^ CFU total) was applied to the apical surface of Calu‐3 monolayers. Co‐cultures were bathed in Krebs salt solution. Normal blood glucose or hyperglycaemia was modelled by applying basolateral glucose concentrations of 5 and 15 mM respectively. Co‐cultures were placed in a CO_2_ incubator at 37°C for 7 hrs, after which either cell lysates were collected for Western blotting or cells were homogenized and CFU calculated by plating out serial dilutions. Bacterial growth was external in the ASL, with minimal epithelial invasion observed after 7 hrs.

### Paracellular L‐glucose flux experiments

Paracellular movement of glucose across Calu‐3 monolayers was measured by analysis of radiolabelled [^14^C]‐L‐glucose transepithelial flux. Experiments were initiated by adding 1 ml of Krebs salt solution containing 1.0 μCi of [^14^C]‐L‐glucose plus 10 mM of non‐radiolabelled equivalent glucose to the basolateral side of the transwells and 0.1 ml of glucose‐free Krebs salt solution to the apical side. Apical and basolateral samples were taken after 1 hr and the concentration of radiolabelled glucose was analysed using a scintillation counter.

### Western blotting for tight junction proteins

After cells were co‐cultured they were lysed in ERK Phosphorylated buffer containing 20 mM Tris‐HCL pH 7.5, 150 mM NaCl, 1 mM ethylenediaminetetraacetic acid, 50 mM NaF, 1 mM NaVO_4_, 1% w/v Triton‐X, 0.5% w/v sodium deoxycholate, 0.1% w/v SDS, 1 μg/ml protease inhibitor cocktail (P8340; Sigma‐Aldrich). Cells were sonicated and cell debris was removed by centrifugation at 10,000 × ***g*** at 4°C for 10 min. Total protein concentration was determined by Bradford Assay and between 30 and 60 μg was electrophoresed on NuPage Novex 10% Bis‐Tris Protein Gels (Invitrogen, Paisley, UK). Protein was then transferred for 70 min. onto polvinylidene difluoride membranes (Merck Millipore, Watford, UK). Non‐specific reactivity was blocked in Odyssey Blocking Buffer diluted 1:1 in PBS for 1 hr at room temperature. Blots were incubated overnight with the following antibodies obtained from Life Technologies and used at 1:500 dilution; mouse anti‐E‐Cadherin (33‐4000), rabbit anti‐Claudin‐1 (51‐9000) and rabbit anti‐Occludin (H‐279; Santa Cruz Biotechnology, Heidelberg, Germany). The blots were then washed in Tris‐buffered saline/0.2% Tween 20 and incubated with either Donkey antimouse IgG (926‐3221; LI‐COR Biosciences, Cambridge, UK) or goat anti‐rabbit IgG (LI‐COR Biosciences) for 1 hr at room temperature and kept in the dark. Blots were subsequently probed with a mouse monoclonal antibody to β‐actin. Detection of antigen–antibody complexes was assessed using a Licor Odyssey Western blot imaging system. Integrated density of each fluorescent band was determined using Image Studio Lite Ver 3.1 (Licor). Results are expressed as a ratio to β‐actin control within the same sample.

### Chemicals and reagents

All chemical and reagents were obtained from Sigma‐Aldrich unless otherwise stated.

### Statistical analysis

Values are reported as mean ± S.E.M. Statistical analysis was performed with anova and post hoc Tukey's or Student's *t*‐test. *P*‐values <0.05 were considered significant.

## Results

### Metformin‐ and AMP‐activated protein kinase agonist AICAR restrict glucose‐induced *P. aeruginosa* growth in airway epithelia‐bacteria co‐cultures

**Figure 1:**
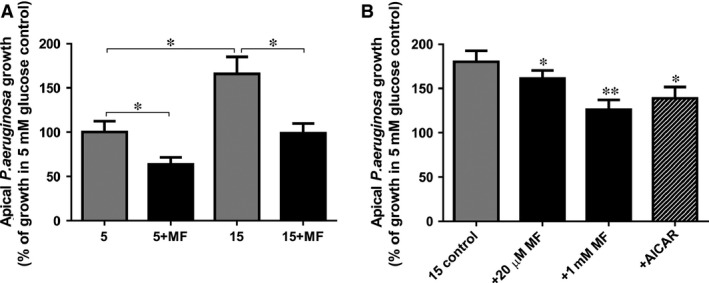
Metformin and AMPK agonist AICAR inhibits hyperglycaemia‐induced *Pseudomonas aeruginosa* growth across Calu‐3 airway epithelial monolayers. (**A**) Airway epithelia‐bacteria co‐cultures grown in the presence of 5 or 15 mM basolateral glucose, without (grey bars) or with pre‐treatment with 1 mM metformin (MF; 18 hr pre‐treatment; black bars). Bacterial CFU were measured 7 hrs after infection (percentage compared with mean growth in the presence of 5 mM basolateral glucose control), *n* = 4, *P < 0.05. (**B**) Airway epithelia‐bacteria co‐cultures grown in the presence of 15 mM basolateral glucose, without (grey bars) or with pre‐treatment with metformin (MF; 20 μM or 1 mM, 18 hr pre‐treatment; black bars) or AICAR (0.5 mM, 18 hr pre‐treatment; hatched bars). Bacterial CFU were measured 7 hrs after infection (percentage compared with mean growth in the presence of 15 mM basolateral glucose control), *n* = 6, *P < 0.05, **P < 0.01 compared to 15 mM basolateral glucose control.

Elevating basolateral glucose from 5 to 15 mM in Calu‐3 airway epithelial co‐cultures increased apical *P. aeruginosa* growth by 66 ± 12% (*P* < 0.01; *n* = 5; Fig. 1A). Pre‐treatment of the epithelium with 1 mM metformin (18 hrs) significantly reduced apical *P. aeruginosa* growth in the presence of normoglycemic (5 mM) and hyperglycaemic (15 mM) basolateral glucose concentrations (*P* < 0.05; *n* = 5). A significant reduction in hyperglycaemic‐induced *P. aeruginosa* growth was also observed using clinically relevant concentrations of metformin (Fig. 1B; 20 μM metformin; usual metformin dose is approximately 15–30 mg/kg/day, producing plasma concentrations ∼5–20 μM) 23, 24.

It is well documented that metformin activates AMP‐activated protein kinase (AMPK) 25, which is known to play an important role in tight junction assembly 26. In addition the AMPK agonist, AICAR has previously been shown to partially prevent tight junction disassembly (induced by calcium depletion) 27. We therefore tested the effect of epithelium pre‐treatment with AICAR (0.5 mM, 18 hrs) and found that this also reduced *P. aeruginosa* growth by 52 ± 9% under hyperglycaemic conditions (*P* < 0.05; *n* = 6; Fig. 1B).

### Metformin attenuates the effects of *P. aeruginosa* on Calu‐3 TEER and transepithelial glucose flux

**Figure 2:**
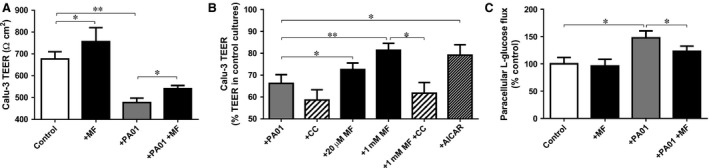
Metformin prevents the effect of *Pseudomonas aeruginosa* infection on transepithelial resistance and paracellular glucose flux across the airway epithelium. (**A**) The effect of metformin pre‐treatment (MF; 1 mM, 18 hrs prior to addition of *P. aeruginosa*) on Calu‐3 monolayer transepithelial electrical resistance (TEER; Ω/cm^2^), in the presence and absence of *P. aeruginosa* (7 hr co‐culture), under normoglycaemic conditions (5 mM basolateral glucose), *n* = 5, *P < 0.05, **P < 0.01. (**B**) The effect of metformin (MF; 20 μM or 1 mM, 18 hr pre‐treatment), AICAR (0.5 mM, 18 hr pre‐treatment) and AMPK inhibitor compound C (CC; 80 μM pre‐treatment for 1 hr prior to addition of metformin) on Calu‐3 monolayer TEER (percentage compared with mean TEER in uninfected control Calu‐3 cultures) in co‐culture with *P. aeruginosa* (7‐hr co‐culture), under normoglycaemic conditions (5 mM basolateral glucose), *n* = 6, *P < 0.05, **P < 0.01. (**C**) Paracellular glucose flux across uninfected and *P. aeruginosa* infected Calu‐3 monolayers, with and without pre‐treatment with 1 mM metformin. Paracellular flux measured by adding radiolabelled L‐glucose to the basolateral surface and monitoring its appearance at the apical surface, *n* = 4, *P < 0.05.

Pre‐treatment with 1 mM metformin increased Calu‐3 monolayer TEER from 676 ± 33 to 756 ± 54 Ω/cm^2^ (*P* < 0.05; *n* = 5; Fig. 2A). The addition of *P. aeruginosa* to the apical surface produced a significant decrease in TEER to 477 ± 21 Ω/cm^2^ after 6 hrs of co‐culture (*P* < 0.01; *n* = 5; Fig. 2A). Metformin treatment (both 1 mM and 20 μM) attenuated the fall in TEER produced by apical *P. aeruginosa* (*P* < 0.05; *n* = 5–6), which was prevented by pre‐treatment with the AMPK inhibitor compound C (Fig. 2B). AICAR pre‐treatment also attenuated the *P. aeuringosa*‐induced reduction in Calu‐3 TEER (*P* < 0.05; *n* = 6; Fig. 2B).

The decrease in TEER evoked by apical *P. aeruginosa* induced an increase in the rate of basolateral‐to‐apical paracellular glucose flux by 47.6 ± 5.8% (*P* < 0.01, *n* = 4; Fig. 2C), which could be partially overcome following metformin treatment (*P* < 0.05; *n* = 4). Metformin had no significant effect on paracellular glucose flux in the absence of *P. aeruginosa* (*P* > 0.05; *n* = 4; Fig. 2C), despite increasing Calu‐3 TEER, consistent with the non‐linear relationship between TEER and paracellular glucose permeability (large changes in TEER are required at high TEER to induce small changes in paracellular flux; conversely small changes in TEER at low TEER produce relatively large changes in paracellular flux) 8.

### Metformin increases claudin‐1 and occludin protein abundance to prevent *P. aeruginosa*‐induced tight junction disruption

We investigated the effects of *P. aeruginosa* and metformin on the abundance of tight junction (claudin‐1 and occludin) and adherens junction (E‐cadherin) proteins.

**Figure 3:**
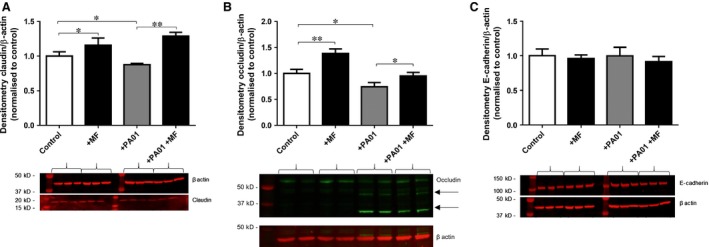
Metformin attenuates *Pseudomonas aeruginosa*‐induced cleavage of epithelial occludin and increases claudin‐1 abundance. Calu‐3 airway epithelial monolayers with and without 1 mM metformin pre‐treatment (18 hrs), were infected with PA01 for 7 hrs, in the presence of 5 mM basolateral glucose. (**A**) Claudin‐1 Western blot band densities normalized against β‐actin control and expressed as comparison to untreated Calu‐3 control lysates, *n* = 4, *P < 0.05, **P < 0.01. (**B**) Occludin Western blot band (65 kD) densities normalized against β‐actin control and expressed as comparison to untreated Calu‐3 control lysates, *n* = 7, *P < 0.05, **P < 0.01. Arrows indicate ~35 and 45 kD occludin cleavage fragments. (**C**) E‐cadherin Western blot band densities normalized against β‐actin control and expressed as comparison to untreated Calu‐3 control lysates, *n* = 3.

Claudin‐1 protein abundance was elevated by 16 ± 8% after 18 hrs in the presence of 1 mM metformin (*P* < 0.05; *n* = 4; Fig. 3). Co‐culture with *P. aeruginosa* produced a small decline in claudin‐1 abundance (*P* < 0.05; *n* = 4), although this could be overcome by metformin pre‐treatment (*P* < 0.01; *n* = 4).

Metformin pre‐treated Calu‐3 cells exhibited significantly increased occludin abundance by 38 ± 9% compared to the untreated controls (*P* < 0.01; *n* = 7; Fig. 3B). *Pseudomonas aeruginosa* addition resulted in reduced abundance of occludin (65 kD band), as well as the appearance of occludin cleavage fragments consistent with previous studies of *P. aeruginosa*‐induced changes in airway epithelia tight junctions 28. Interestingly, the abundance of the *P. aeruginosa*‐induced occludin cleavage fragments (~45 kD and ~35 kD) was significantly reduced by metformin pre‐treatment (by 34 ± 7% and 18 ± 5%, respectively; *P* < 0.05; *n* = 4).

E‐cadherin abundance was not significantly altered by either metformin or *P. aeruginosa* addition (*P* > 0.05; *n* = 3; Fig. 3C).

## Discussion

Our study highlights the potential of metformin to reduce hyperglycaemia‐induced *P. aeruginosa* growth through modulation of airway epithelial tight junctions. Metformin pre‐treatment of Calu‐3 airway epithelial cells reduced hyperglycaemia‐induced apical *P. aeruginosa* growth consistent with our previous observations where metformin decreased hyperglycaemia‐induced apical *S. aureus* growth on H441 and primary human bronchial epithelial cells (HBEC) 12. A similar attenuation of *P. aeruginosa*‐induced changes in TEER and restricted bacterial growth were observed with the AMPK agonist, AICAR and metformin failed to significantly alter the bacteria‐induced reduction in TEER in the presence of the AMPK inhibitor, compound C. These data therefore indicate a potential AMPK‐dependence in metformin's ability to prevent *P. aeruginosa*‐induced impairment of airway epithelial barrier function.

Metformin increased occludin and claudin‐1 abundance which correlated with increased TEER and reduced paracellular glucose flux across Calu‐3 cells. The route by which glucose crosses airway epithelial tight junctions has not been defined. We have shown that, although glucose permeability is related to TEER which is a measurement of ionic permeability it is not a direct linear relationship 8. Moreover, permeability of ions can be restricted by the presence of specific tight junction proteins which influence charge selectivity of the tight junction 29. Thus, the alterations in tight junction composition that determine glucose permeability as a larger non‐ionic solute are likely to be different to that of ions. Exploring the tight junction proteins modified by metformin to reduce glucose permeability in the presence of bacteria may highlight key proteins involved in regulating paracellular glucose movement.

*Pseudomonas aeruginosa* addition reduced the abundance of full‐length occludin (~65 kD) and produced occludin‐cleavage products consistent with previous reports 28. Furthermore, we showed that this was associated with increased glucose permeability confirming previous findings that reduced abundance of occludin increased the permeability of epithelial cells to ethanolamine and mannitol, non‐charged solutes of similar size to glucose 30.

Cleavage of occludin was shown to occur *via* TLR‐2‐dependent activation of calpains in airway epithelial cell monolayers co‐cultured with *P. aeruginosa* strain PA01 28. The abundance of cleaved occludin proteins was significantly reduced by metformin, suggesting that metformin may inhibit this pathway. Metformin is known to have anti‐inflammatory effects in airway epithelial cells 31 and in a study of 30 diabetic patients, those on metformin therapy exhibited lower blood TLR‐2 expression 32. Calpains are Ca^2+^‐dependent proteases that are ubiquitously expressed in cells and tissues. Knockdown of calpains 1 and 2 reduced cleavage of occludin in epithelial cells 28. Currently, there is no evidence for any effect of metformin on calpain activity in airway cells although metformin treatment efficacy in patients has been linked with polymorphisms in the gene for calpain‐10 33.

Claudin‐1 abundance was also reduced in the presence of *P. aeruginosa*, which has similarly been shown to be regulated by TLR2‐dependent signalling pathways in Calu‐3 cells 34. Claudin‐1 is expressed ubiquitously in most epithelial tissues of the body 35, including the airway epithelium and to a lesser extent the alveolar epithelium 36, 37. *In vitro* studies have shown that claudin‐1 acts predominantly as a barrier‐forming tight junction protein, with overexpression leading to an increase in TEER 38, 39. Knockout of claudin‐1 in mice leads to loss of the tight junctional barrier to water and macromolecules across the epidermis 40. Consistent with our findings, downregulation of claudin‐1 increased paracellular permeability to non‐charged molecules in airway epithelial cells 41.

Interestingly, TLR2 agonists were able to modify claudin‐1 expression through atypical PKC ζ 34, which is a known target of AMPK in alveolar epithelial cells 42. Given that metformin is an AMPK agonist, and similar effects on TEER and bacterial growth were produced by the AMPK agonist AICAR, it is possible that claudin‐1 expression is regulated by metformin through AMPK‐dependent phosphorylation of PKC ζ.

The changes in abundance shown in this study highlight potential roles for claudin‐1 and occludin in the action of metformin to prevent the *P. aeruginosa*‐induced increase in glucose permeability and hyperglycaemia‐induced growth. Although metformin treatment is likely to affect other tight junction proteins, both claudin‐1 and occludin could be important targets to reduce glucose permeability across airway epithelia and supress bacterial growth. Further investigation into the mechanisms regulating metformin and *P. aeruginosa* action on airway epithelial tight junctions are now required. Such studies could yield potential alternatives to antibiotics to prevent/suppress hyperglycaemia‐induced respiratory infections.

## Conflicts of interest

The authors confirm that there are no conflicts of interest.

## Author contribution

DLB, EHB and JPG designed the research study; WP, GC and JPG performed the research; WP, GC, DLB and JPG analysed the data; DLB and JPG wrote the paper. GC contributed to revision of the manuscript.

## References

[1] Baker EH , Clark N , Brennan AL , *et al* Hyperglycemia and cystic fibrosis alter respiratory fluid glucose concentrations estimated by breath condensate analysis. J Appl Physiol. 2007; 102: 1969–75. 17303703 10.1152/japplphysiol.01425.2006

[2] Philips BJ , Meguer JX , Redman J , *et al* Factors determining the appearance of glucose in upper and lower respiratory tract secretions. Intensive Care Med. 2003; 29: 2204–10. 14647890 10.1007/s00134-003-1961-2

[3] Lee RJ , Kofonow JM , Rosen PL , *et al* Bitter and sweet taste receptors regulate human upper respiratory innate immunity. J Clin Invest. 2014; 124: 1393–405. 24531552 10.1172/JCI72094 PMC3934184

[4] Baker EH , Janaway CH , Philips BJ , *et al* Hyperglycaemia is associated with poor outcomes in patients admitted to hospital with acute exacerbations of chronic obstructive pulmonary disease. Thorax. 2006; 61: 284–9. 16449265 10.1136/thx.2005.051029 PMC2104606

[5] Wood DM , Brennan AL , Philips BJ , *et al* Effect of hyperglycaemia on glucose concentration of human nasal secretions. Clin Sci. 2004; 106: 527–33. 14678009 10.1042/CS20030333

[6] Brennan AL , Gyi KM , Wood DM , *et al* Airway glucose concentrations and effect on growth of respiratory pathogens in cystic fibrosis. J Cyst Fibros. 2007; 6: 101–9. 16844431 10.1016/j.jcf.2006.03.009

[7] Philips BJ , Redman J , Brennan A , *et al* Glucose in bronchial aspirates increases the risk of respiratory MRSA in intubated patients. Thorax. 2005; 60: 761–4. 16135681 10.1136/thx.2004.035766 PMC1747508

[8] Kalsi KK , Baker EH , Fraser O , *et al* Glucose homeostasis across human airway epithelial cell monolayers: role of diffusion, transport and metabolism. Pflugers Arch. 2009; 457: 1061–70. 18781323 10.1007/s00424-008-0576-4

[9] Garnett JP , Nguyen TT , Moffatt JD , *et al* Proinflammatory mediators disrupt glucose homeostasis in airway surface liquid. J Immunol. 2012; 189: 373–80. 22623330 10.4049/jimmunol.1200718 PMC3605804

[10] Garnett JP , Baker EH , Baines DL . Sweet talk ‐ insights into the nature & importance of glucose transport in lung epithelium. Eur Respir J. 2012; 40: 1269–76. 22878875 10.1183/09031936.00052612

[11] Garnett JP , Braun D , McCarthy AJ , *et al* Fructose transport‐deficient *Staphylococcus aureus* reveals important role of epithelial glucose transporters in limiting sugar‐driven bacterial growth in airway surface liquid. Cell Mol Life Sci. 2014; 71: 4665–73. 24810961 10.1007/s00018-014-1635-y PMC4232747

[12] Garnett JP , Baker EH , Naik S , *et al* Metformin reduces airway glucose permeability and hyperglycaemia‐induced *Staphylococcus aureus* load independently of effects on blood glucose. Thorax. 2013; 68: 835–45. 23709760 10.1136/thoraxjnl-2012-203178 PMC3756442

[13] Garnett JP , Gray MA , Tarran R , *et al* Elevated paracellular glucose flux across cystic fibrosis airway epithelial monolayers is an important factor for *Pseudomonas aeruginosa* growth. PLoS ONE. 2013; 8: e76283. 24124542 10.1371/journal.pone.0076283 PMC3790714

[14] Shen BQ , Finkbeiner WE , Wine JJ , *et al* Calu‐3: a human airway epithelial cell line that shows cAMP‐dependent Cl‐ secretion. Am J Physiol. 1994; 266: L493–501. 7515578 10.1152/ajplung.1994.266.5.L493

[15] Kreda SM , Okada SF , van Heusden CA , *et al* Coordinated release of nucleotides and mucin from human airway epithelial Calu‐3 cells. J Physiol. 2007; 584: 245–59. 17656429 10.1113/jphysiol.2007.139840 PMC2277076

[16] Joo NS , Lee DJ , Winges KM , *et al* Regulation of antiprotease and antimicrobial protein secretion by airway submucosal gland serous cells. J Biol Chem. 2004; 279: 38854–60. 15234967 10.1074/jbc.M407077200

[17] Grainger CI , Greenwell LL , Lockley DJ , *et al* Culture of Calu‐3 cells at the air interface provides a representative model of the airway epithelial barrier. Pharm Res. 2006; 23: 1482–90. 16779708 10.1007/s11095-006-0255-0

[18] Wan H , Winton HL , Soeller C , *et al* Tight junction properties of the immortalized human bronchial epithelial cell lines Calu‐3 and 16HBE14o‐. Eur Respir J. 2000; 15: 1058–68. 10885425 10.1034/j.1399-3003.2000.01514.x

[19] Garnett JP , Hickman E , Burrows R , *et al* Novel role for pendrin in orchestrating bicarbonate secretion in cystic fibrosis transmembrane conductance regulator (CFTR)‐expressing airway serous cells. J Biol Chem. 2011; 286: 41069–82. 21914796 10.1074/jbc.M111.266734 PMC3220502

[20] Salomon JJ , Endter S , Tachon G , *et al* Transport of the fluorescent organic cation 4‐(4‐(dimethylamino)styryl)‐*N*‐methylpyridinium iodide (ASP+) in human respiratory epithelial cells. Eur J Pharm Biopharm. 2012; 81: 351–9. 22426135 10.1016/j.ejpb.2012.03.001

[21] Ingoglia F , Visigalli R , Rotoli BM , *et al* Functional characterization of the organic cation transporters (OCTs) in human airway pulmonary epithelial cells. Biochim Biophys Acta. 2015; 1848: 1563–72. 25883089 10.1016/j.bbamem.2015.04.001

[22] Chen L , Pawlikowski B , Schlessinger A , *et al* Role of organic cation transporter 3 (SLC22A3) and its missense variants in the pharmacologic action of metformin. Pharmacogenet Genomics. 2010; 20: 687–99. 20859243 10.1097/FPC.0b013e32833fe789 PMC2976715

[23] Sum CF , Webster JM , Johnson AB , *et al* The effect of intravenous metformin on glucose metabolism during hyperglycaemia in type 2 diabetes. Diabet Med. 1992; 9: 61–5. 1551312 10.1111/j.1464-5491.1992.tb01716.x

[24] Radwan MA , Al Taweel ES , Al‐Moghairi AM , *et al* Monitoring metformin in cardiac patients exposed to contrast media using ultra‐high‐performance liquid chromatography tandem mass‐spectrometry. Ther Drug Monit. 2011; 33: 742–9. 22105592 10.1097/FTD.0b013e318237ab03

[25] Zhou G , Myers R , Li Y , *et al* Role of AMP‐activated protein kinase in mechanism of metformin action. J Clin Invest. 2001; 108: 1167–74. 11602624 10.1172/JCI13505 PMC209533

[26] Zhang L , Li J , Young LH , *et al* AMP‐activated protein kinase regulates the assembly of epithelial tight junctions. Proc Natl Acad Sci USA. 2006; 103: 17272–7. 17088526 10.1073/pnas.0608531103 PMC1859922

[27] Zheng B , Cantley LC . Regulation of epithelial tight junction assembly and disassembly by AMP‐activated protein kinase. Proc Natl Acad Sci USA. 2007; 104: 819–22. 17204563 10.1073/pnas.0610157104 PMC1783397

[28] Chun J , Prince A . TLR2‐induced calpain cleavage of epithelial junctional proteins facilitates leukocyte transmigration. Cell Host Microbe. 2009; 5: 47–58. 19154987 10.1016/j.chom.2008.11.009 PMC2768384

[29] Anderson JM , Van Itallie CM . Physiology and function of the tight junction. Cold Spring Harb Perspect Biol. 2009; 1: a002584. 20066090 10.1101/cshperspect.a002584 PMC2742087

[30] Yu AS , McCarthy KM , Francis SA , *et al* Knockdown of occludin expression leads to diverse phenotypic alterations in epithelial cells. Am J Physiol Cell Physiol. 2005; 288: C1231–41. 15689410 10.1152/ajpcell.00581.2004

[31] Myerburg MM , King JD Jr , Oyster NM , *et al* AMPK agonists ameliorate sodium and fluid transport and inflammation in cystic fibrosis airway epithelial cells. Am J Respir Cell Mol Biol. 2010; 42: 676–84. 19617399 10.1165/rcmb.2009-0147OC PMC2891496

[32] Andrews M , Soto N , Arredondo M . Effect of metformin on the expression of tumor necrosis factor‐α, Toll like receptors 2/4 and C reactive protein in obese type‐2 diabetic patients. Rev Med Chil. 2012; 140: 1377–82. 23677182 10.4067/S0034-98872012001100001

[33] Tkáč I , Javorský M , Klimčáková L , *et al* A pharmacogenetic association between a variation in calpain 10 (CAPN10) gene and the response to metformin treatment in patients with type 2 diabetes. Eur J Clin Pharmacol. 2015; 71: 59–63. 25327507 10.1007/s00228-014-1774-y

[34] Ragupathy S , Esmaeili F , Paschoud S , *et al* Toll‐like receptor 2 regulates the barrier function of human bronchial epithelial monolayers through atypical protein kinase C zeta, and an increase in expression of claudin‐1. Tissue Barriers. 2014; 2: e29166. 25101232 10.4161/tisb.29166 PMC4117686

[35] Furuse M , Fujita K , Hiiragi T , *et al* Claudin‐1 and ‐2: novel integral membrane proteins localizing at tight junctions with no sequence similarity to occludin. J Cell Biol. 1998; 141: 1539–50. 9647647 10.1083/jcb.141.7.1539 PMC2132999

[36] Chen SP , Zhou B , Willis BC , *et al* Effects of transdifferentiation and EGF on claudin isoform expression in alveolar epithelial cells. J Appl Physiol. 2005; 98: 322–8. 15361518 10.1152/japplphysiol.00681.2004

[37] Wang F , Daugherty B , Keise LL , *et al* Heterogeneity of claudin expression by alveolar epithelial cells. Am J Respir Cell Mol Biol. 2003; 29: 62–70. 12600828 10.1165/rcmb.2002-0180OC

[38] Inai T , Kobayashi J , Shibata Y . Claudin‐1 contributes to the epithelial barrier function in MDCK cells. Eur J Cell Biol. 1999; 78: 849–55. 10669103 10.1016/S0171-9335(99)80086-7

[39] McCarthy KM , Francis SA , McCormack JM , *et al* Inducible expression of claudin‐1‐myc but not occludin‐VSV‐G results in aberrant tight junction strand formation in MDCK cells. J Cell Sci. 2000; 113: 3387–98. 10984430 10.1242/jcs.113.19.3387

[40] Furuse M , Hata M , Furuse K , *et al* Claudin‐based tight junctions are crucial for the mammalian epidermal barrier: a lesson from claudin‐1‐deficient mice. J Cell Biol. 2002; 156: 1099–111. 11889141 10.1083/jcb.200110122 PMC2173463

[41] Gan H , Wang G , Hao Q , *et al* Protein kinase D promotes airway epithelial barrier dysfunction and permeability through down‐regulation of claudin‐1. J Biol Chem. 2013; 288: 37343–54. 24265314 10.1074/jbc.M113.511527 PMC3873586

[42] Gusarova GA , Dada LA , Kelly AM , *et al* Alpha1‐AMP‐activated protein kinase regulates hypoxia‐induced Na, K‐ATPase endocytosis *via* direct phosphorylation of protein kinase C zeta. Mol Cell Biol. 2009; 29: 3455–64. 19380482 10.1128/MCB.00054-09 PMC2698765

